# Tooth Mobility among Patients Visiting a Tertiary Care Centre: A Descriptive Cross-sectional Study

**DOI:** 10.31729/jnma.7945

**Published:** 2023-01-31

**Authors:** Sujaya Gupta, Sangya Mukhiya, Anuja Kafle, Subina Ghimire, Usha Kiran Acharya

**Affiliations:** 1 Department of Periodontics and Oral Implantology, Kathmandu Medical College and Teaching Hospital, Duwakot, Bhaktapur, Nepal

**Keywords:** *periodontitis*, *prevalence*, *tooth mobility*

## Abstract

**Introduction:**

Periodontitis is a chronic inflammatory disease that results in the destruction of supporting tissue and bone leading to tooth mobility. Tooth mobility if untreated can lead to tooth loss. However, very few studies exist for its assessment. The aim of this study was to find out the prevalence of tooth mobility among patients visiting a tertiary care centre.

**Methods:**

This descriptive cross-sectional study was conducted among individuals visiting a tertiary care dental hospital from 1 April to 30 June 2022 after obtaining ethical clearance from the Institutional Review Committee (Reference number: 2202202202). Individuals more than 13 years who gave consent and fulfilled the study criteria were enrolled. Tooth mobility was assessed using Lindhe and Nyman's classification. Proforma also included demographics, simplified oral hygiene index, gingival index, body mass index, and smoking status. Convenience sampling was done. Point estimate and 95% Confidence Interval were calculated.

**Results:**

Among 163 patients, 65 (39.88%) patients (32.36-47.40, 95% Confidence Interval) had tooth mobility.

**Conclusions:**

The prevalence of tooth mobility was higher than in studies done in similar settings.

## INTRODUCTION

Tooth mobility is the non-physiological horizontal and, to a lesser degree, axial movement of a tooth in response to normal forces, as in occlusion, resulting from loss of all or a portion of its attachment, and supportive apparatus.^1,2^ It is an important feature of periodontal disease, which begins as gingivitis progresses to affect the periodontal ligament, leading to bone loss, tooth mobility, and the ultimate loss of teeth if left untreated.^1^

Tooth mobility is one criterion to evaluate the status of the periodontium. It has become an important diagnostic parameter for clinicians to determine the integrity, functional state, and disease of periodontium.^3^ Hence, its examination is important in planning dental treatment, as it may give an indication of alveolar bone loss and the condition of the periodontal ligament.

The aim of this study was to find out the prevalence of tooth mobility among patients visiting a tertiary care centre.

## METHODS

A descriptive cross-sectional study was conducted in the dental hospital of Kathmandu Medical College and Teaching Hospital (KMCTH). The study was conducted for 3 months from 1 April 2022 to 30 June 2022 after obtaining ethical clearance from the Institutional Review Committee (Reference number: 2202202202). Informed consent was obtained before conducting the interview and oral examination.

The inclusion criteria included individuals more than 13 years of age, visiting the teaching hospital of KMCTH for any reason and willing to participate in the study. The exclusion criteria were: pregnant and lactating women, patients undergoing orthodontic treatment, patients who have recently (within the past 3 months) undergone periodontal therapy, patients with exfoliative mobility, patients with mobile root stump(s), patients with mobile third molars; and individuals with tooth mobility due to trauma.

The sample size was been calculated using the following formula:


$$\begin{array}{ll} \text{n} & ={\text{Z}}^{2}\times \frac{\text{p}\times \text{q}}{{\text{e}}^{\text{2}}}\\ & =1.96^{2}\times \frac{0.102\times 0.898}{0.5^{2}}\\ & =141 \end{array}$$

Where,

n = minimum required sample size
Z = 1.96 at 95 % Confidence Interval (CI)
p = prevalence taken from previous studies, 10.2%^4^
q = 1-p
e = margin of error, 5%

Adding 10% non-response rate, the sample size came as 155. Hence, the total number of participants taken was 163.

The severity of tooth mobility was assessed based on the classification of tooth mobility given by Lindhe and Nyman (1975)^5,6^ also referred to as Nyman's Index (1975) by some.^2,7-9^

Smoking behaviour was categorised as current smokers (smoked ≥100 cigarettes in their lifetime and currently smoke), former smokers (smoked ≥100 cigarettes in their lifetime and do not currently smoke), and non-smokers (not smoked ≥100 cigarettes in their lifetime and do not currently smoke).^10^

The oral hygiene status was assessed using Greene and Vermillion's Simplified Oral Hygiene Index (OHI-S)^11^ followed by assessing the severity of gingivitis using the Gingival Index (GI) developed by Loe and Silness (1963).^12^

Data were analysed using IBM SPSS Statistics version 20.0 and results were tabulated in Microsoft Excel 2016. Point estimate and 95% Confidence Interval were calculated.

## RESULTS

Of the 163 participants examined, 65 (39.88%) (32.3647.40, 95% Confidence Interval) had tooth mobility.

Out of 65 tooth mobility patients, 37 (56.92%) females had the most tooth mobility. Higher tooth mobility was seen in the elderly group 32 (49.23%). Nine (13.85%) participants had visited any dental hospital or doctor for the very first time. Forty (61.54%) had irregular brushing habits (Table 1). Twenty (30.77%) of the participants were homemakers.

**Table 1:** Different parameters of tooth mobility (n= 65).

n (%)		
Gender	Male	28 (43.08)
	Female	37 (56.92)
Age (years)	Adolescent (≤25)	9 (13.85)
	Adult (26-45)	24 (36.92)
	Elderly (≥46)	32 (49.23)
BMI (kg/m^2^)	Underweight (<18.5)	2 (3.08)
	Normal weight (18.5 to <25)	28 (43.08)
	Overweight (25 to <30)	33 (50.77)
	Obese (≥30)	2 (3.08)
Brushing frequency	No brushing	-
	Irregular (≤1/day)	40 (61.54)
	Regular (≥2/day)	25 (38.46)
Toothpaste	Fluoridated	46 (70.77)
	Non-fluoridated	19 (29.23)
Softness of toothbrush	Don't know	3 (4.62)
	Hard	2 (3.08)
	Medium	24 (36.92)
	Soft	36 (55.38)
Smoking habit	Current smoker	10 (15.38)
	Former smoker	12 (18.46)
	Non-smoker	43 (66.15)
Gingival	Mild gingivitis	4 (6.15)
index (Loe and Silness)	Moderate gingivitis	46 (70.77)
	Severe gingivitis	15 (23.08)
Simplified oral hygiene index (OHI-S)	Good	2 (3.08)
	Fair	20 (30.08)
	Poor	43 (66.15)

The mean age of the participants was 42.72±13.14 years and the mean gingival index score was 1.92±0.56 (Table 2).

**Table 2:** Parameters of tooth mobility (n= 65).

Parameters	Mean±SD
Number of teeth present	27.29±1.28
Age (years)	42.72±13.14
Body mass index (kg/m^2^)	25.56±5.34
Number of cigarettes smoked	8.20±5.29
Number of years smoked	14.70±10.51
Pack years of smoking	6.41±6.13
Gingival index score	1.92±0.56
Simplified debris index score	1.85±0.63
Simplified calculus index score	1.81±0.75
Simplified oral hygiene index score	3.65±1.31

Upon assessing the severity of tooth mobility, Degree 1 mobility was observed in all 65 (100%) participants with tooth mobility. The Degree 2 tooth mobility was seen in 14 (21.54%), and Degree 3 in only 3 (4.62%) participants (Table 3).

**Table 3:** The severity of tooth mobility according to gender and age (n= 65).

Parameters		Degree 1 n (%)	Degree 2 n (%)	Degree 3 n (%)
Gender	Male	28 (43.08)	5 (7.69)	1 (1.53)
	Female	37 (56.92)	9 (13.85)	2 (3.08)
Age	Adolescent (≤25)	9 (13.85)	1 (1.53)	-
	Adult (26-45)	24 (36.92)	1 (1.53)	1 (1.53)
	Elderly and senior (≥46)	32 (49.23)	12 (18.46)	2 (3.08)

Among 65 participants with tooth mobility, 271 teeth were mobile. Out of 271 mobile teeth, 234 (86.35%) were Degree 1 mobile. The most commonly involved teeth were in the mandibular anterior region with 196 (72.32%), followed by 34 (12.55%) in the maxillary anterior, 23 (8.49%) in the mandibular posterior, and the least 18 (6.64%) in the maxillary posterior region (Table 4).

**Table 4:** Maternal demographics (n= 31).

Tooth type (two-digit numbering system)	Degree 1	Degree 2	Degree 3	Total	Location	n (%)
17	2			2	Maxillary posterior	8 (2.95)
16	1			1		
15	2			2		
14	3			3		
13					Maxillary anterior	34(12.55)
12	4			4		
11	8	1		9		
21	12			12		
22	7	2		9		
23						
24	4	1		5	Maxillary posterior	10(3.69)
25	3	1		4		
26						
27	1			1		
37	5			5	Mandibular posterior	16(5.90)
36	4	1		5		
35	3	1		4		
34	1	1		2		
33	4	2		6	Mandibular anterior	196 (72.32)
32	40	4	1	45		
31	44	6	2	52		
41	45	7	2	54		
42	28	4		32		
43	6	1		7		
44	3			3	Mandibular	7 (2.58)
45					posteriors	
46	3			3		
47	1			1		
Total	234 (86.35)	32 (11.81)	5 (1.84)	271 (100)		271

Among the 65 participants with tooth mobility, 19 (29.23%) had underlying systemic conditions where with 11 (16.92%) with hypertension forming the majority, 5 (7.69%) had diabetes mellitus, 2 (3.08%) hyperthyroidism, 2 (3.08%) hypothyroidism, and 1 (1.54%) reported asthma, hypercholesterolaemia, and hemiplegia each. Rest 46 (70.77%) reported no underlying systemic condition.

## DISCUSSION

Periodontal disease is one of the most common oral diseases. A healthy periodontium is vital for the maintenance of teeth in the oral cavity. The initial stage of gingivitis progresses in a sequential manner towards periodontitis ultimately resulting in tooth loss. Thus, if tooth mobility is identified and treated at an early stage, tooth loss can be prevented.

In this study, out of the 163 participants, 65 (39.88%) had tooth mobility. However, a recent study^4^ reported only 10.2% and another study^1^ reported 18.8% prevalence in Nigerian population. The difference could be because the first cited study was a retrospective study done in a Specialist Periodontology clinic and the second cited study was done in a rural population, both with Miller's index. The current study was done in the general dental hospital with a small sample size, short study duration and using Lindhe and Nyman's index because it is more specific and clearly defined than Miller's classification.^5^ The 39.88% prevalence signifies that tooth mobility is a quite common health problem in the studied sample of the Nepali population. Thus, tooth mobility if left untreated, will lead to loss of tooth disturbing the occlusal balance, difficulty in chewing food, and aesthetic problems.

Females 37 (56.92%) exhibited more tooth mobility than males 28 (43.08%) in the present study. It may be due to the influence of female sex hormones. Similar findings were reported in Greek^8^ and Nigerian^4^ populations, however, males had more tooth loss in a study of the Indian^13^ population.

Like previous studies, tooth mobility was observed more commonly in anterior teeth.^4,8,14^ Out of 271 mobile teeth, 196 (72.32%) were of mandibular anterior region. The increased resistance of posterior teeth relative to anterior teeth to tooth mobility may be due to the increased number and divergence of roots of posterior teeth. The mandibular teeth also erupt ahead of maxillary teeth therefore they have the likelihood to succumb to the cumulative destructive effect of insults from oral deposits, more specifically plaque explaining why mobile teeth were more in mandibular than the maxillary arch. The likelihood of anterior tooth mobility followed by its loss, adversely affects oral health-related quality of life, thus impacting daily life and satisfaction.^15,16^

The mean DI-S, CI-S, and OHI-S scores were 1.85±0.63; 1.81±0.75; and 3.65±1.31 respectively. This was more when compared with a study done in the Nigerian population.^4^ It is slightly puzzling as the brushing frequency of the current study (brushing frequency ≥2/ day = 25, 38.46%) was similar to the previous study of 38.7%. This might imply that the participants of this study might not be following proper brushing techniques and could need guidance and counselling regarding the same. However, in current as well as previous studies all the GI-S, DI-S, CI-S and OHI-S scores were more in the individuals with tooth mobility.^1,4^

Regarding dental visits, 9 (13.85%) had never visited a dentist leading to the rest 56 (86.15%) who had visited at least once. This was better than that reported in a Nigerian study which reported that for 41.3% of participants it was their first dental visit.^4^ Though this shows Nepali population could be more aware of their oral health compared to the Nigerian population, it is still not up to par. Although there is a lack of adequate supporting research, most dental associations throughout the world recommend all to visit dentists, once every 6 months and no later than 2 years.

A total of 271 teeth were found to be mobile in 65 participants in the current study. Out of 271 mobile teeth, 234 (86.35%) were Degree 1 mobile. This was similar to that reported in the Indian population.^14^ Similar findings were reported in the Greek population as well.^8^ However, it was different than that reported in Nigerian individuals where only 52% were Degree 1 and rest were Degree 2, and Degree 3.^4^ It could be because of more elderly and senior population in the latter study.

Of all participants with tooth mobility, only 19 (29.23%) reported having underlying systemic conditions. This is in contrast to that reported among the Nigerian population where nearly half (48.00%) had some sort of underlying medical condition. The difference could be because of differences in population and age group. The current study was done in the Nepali population which had 32 (48.23%) individuals in the "elderly and senior" age group. Regarding BMI and tooth mobility, more individuals were in the "overweight" category, followed immediately by the normal weight category. Only 2 (3.08%) were in the obese or underweight categories. It could be because of the small sample size. Tobacco smoking is a proven risk factor for periodontal disease and is associated with increased severity.^10,17^ Similarly, all three parameters of smoking recorded: number of cigarettes per day, number of years smoked, and pack years of smoking, were raised in the participants with tooth mobility. This is similar to previous studies.^8,18,19^ However, the finding should be taken cautiously as most 43 (66.15%) participants were non-smokers in this study. Furthermore, it could also imply that the participants did not accurately report their true smoking status.^18,20^

The retention of the teeth is a major goal of dental treatment.^13^ Mobility of tooth, the precursor of tooth loss, is directly related to the loss of clinical periodontal attachment, pocket depth, and radiographic bone loss.^8^ Hence, early diagnosis, prevention, and management of tooth mobility becomes vital for the optimum oral and periodontal health.

The limitations of this study were that it was a singlecentre descriptive cross-sectional study with small sample size and short duration. Since the GI and OHI-S measurements were done only in the index teeth, it can overestimate or underestimate the prevalence of periodontal disease and oral hygiene status respectively.

## CONCLUSIONS

The prevalence of tooth mobility was higher than in studies done in similar settings. The prevalence was observed in more than one-third of the participants. Though the majority had only Degree 1 mobile teeth, it still is a cause for concern. Hence to prevent tooth loss that leads to poor masticatory efficiency, the Nepali population should be made more aware of different measures for better oral and periodontal health. Tooth mobility despite being such an important parameter for the assessment of the severity of periodontal disease and prediction for tooth loss, the authors could find very few studies. Hence, multicentric studies with more sample sizes are highly recommended.
